# Exploring lipid diversity and minimalism to define membrane requirements for synthetic cells

**DOI:** 10.1002/1873-3468.70131

**Published:** 2025-08-11

**Authors:** Sergiy Gan, Victoria Scarpelli, Marten Exterkate

**Affiliations:** ^1^ Membrane Biogenesis and Lipidomics group, Institute of Biochemistry Heinrich Heine University Düsseldorf Germany

**Keywords:** bottom‐up synthetic biology, compartmentalization, JCVI‐Syn3A, lipid synthesis, synthetic cell

## Abstract

The creation of minimal synthetic cells that mimic the essential functions of biological cells is a long‐term goal in synthetic biology. Achieving this objective not only advances our understanding of the origin of life, but also unlocks the way for applications in industry, medicine, etc. A key characteristic of life is self‐reproduction, which includes growth and division of the cell and its membrane. This boundary layer is formed by a lipid matrix in which proteins are anchored. The complexity of natural lipid membranes is a major challenge for the construction of a minimal system, as it directly influences membrane shape and protein function. Although simple synthetic compartmentalization systems can consist of a single lipid species, there is substantial uncertainty regarding the complexity of the lipidome required to sustain the essential functions of a self‐reproducing cell. This Review highlights the contrast between bottom‐up and top‐down approaches toward synthetic cell construction, emphasizing the critical interplay between membrane proteins and their surrounding lipid environment. We explore the complexity and compatibility of membrane systems and discuss minimal lipidome requirements for synthetic cellular systems.

**Impact statement**

Synthetic cell research will help us to truly understand the basic principles of cellular life, in which lipid membranes are crucial. Ultimately, synthetic cells should lead to engineered specialized entities that can be applied in various fields, including medicine, bio‐technology, and environmental science.

## Graphical abstract

Designing the lipid membrane of synthetic cells is a complex task, in which its various roles (among them solute transport, membrane protein support, and self‐replication) should all be integrated. In this review, we report the latest top‐down and bottom‐up advances and discuss compatibility and complexity issues of current engineering approaches toward a synthetic cellular lipid membrane.

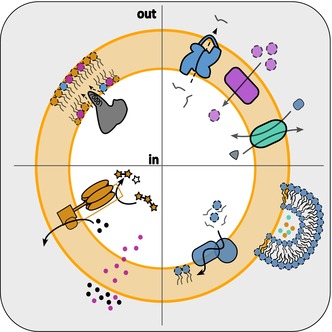

## Abbreviation

**AAC**, ADP/ATP carrier protein

***ABC***, cardiolipin biosynthesis genes ABC

**ACP**, acyl carrier protein

***Arc*ABCD**, arginine deiminase pathway gene cluster

**AX**, archaeol phosphate with variable headgroup X

**AY**, archaeol with variable headgroup Y

***B. subtilis***, *Bacillus subtilis*

**b**_**6**_ **c:caa**_**3**_, Multi‐enzyme complex cytochrome b_6_c with cytochrome caa_3_ oxidase

**Bbp**, billion base pair

**CDP‐archaeol**, cytidine diphosphate archaeol

**CDP‐DAG**, cytidine diphosphate diacylglycerol

**CdsA**, Phosphatidate cytidylyltransferase

**CerS**, ceramide synthase

**CL**, cardiolipin

**CMP**, cytidine monophosphate

**CoA**, coenzyme A

**CTP**, cytidine triphosphate

**D.PC**, diether 16:0–18:1 phosphatidylcholine

**DAG**, diacylglycerol

**DGGGP**, digeranylgeranylglyceryl phosphate

**DNA**, deoxyribonucleic acid

**DOPE**, 1,2‐dioleoyl‐sn‐glycero‐3‐phosphoethanolamine

**DOPG**, 1,2‐dioleoyl‐sn‐glycero‐3‐phosphatidylglycerol

**DPPE**, 1,2‐dipalmitoyl‐sn‐glycero‐3‐phosphoethanolamine

**DPPG**, 1,2‐dipalmitoyl‐sn‐glycero‐3‐phosphatidylglycerol

**DSPE‐PEG**, 1,2‐distearoyl‐sn‐glycero‐3‐phosphoethanolamine‐polyethylene glycol

***E. coli***, *Escherichia coli*

**FA**, fatty acids

**FAAs**, fatty acyl adenylates

**FadD**, Long‐chain acyl‐CoA synthetase

**FASII**, multi‐enzyme protein fatty acid synthase

**FtsZ**, cell division protein FtsZ

**G1P**, glycerol‐1‐phosphate

**G3P**, glycerol‐3‐phosphate

**GGGP**, geranylgeranylglyceryl phosphate

**GGPP**, geranylgeranyl diphosphate

**GlpK**, glycerol kinase

**GlpT**, glycerol‐3‐phosphate/phosphate antiporter

**Glycerol‐P**, glycerol‐phosphate

**GPAT**, glycerol‐phosphate acyltransferase

**GUV**, giant unilamellar vesicle

**JCVI‐syn3.0**, John Craig Venter Institute – synthetic version 3.0

**Kbp**, kilobase pairs

**LacY**, lactose‐proton symporter

**LB**, lysogeny broth

**LPA**, lysophosphatidic acid

**LPAAT**, lysophosphatidic acid acyltransferase

**LPG**, lysyl‐phosphatidylglycerol

**LPS**, lipopolysaccharide

***M. mycoides***, *Mycoplasma mycoides*

**Mbp**, megabase pairs

**NHPI**, N‐hydroxyphtalimide

**OpuA**, glycine betaine transport ATP‐binding protein

**PA**, phosphatidic acid

**PC**, phosphatidylcholine

**Pds**, phosphatidylserine decarboxylase proenzyme

**PE**, phosphatidylethanolamine

**PG**, phosphatidylglycerol

**P‐gp**, P‐glycoprotein

**PgpA**, phosphatidylglycerolphosphate phosphatase A

**PgsA**, phosphatidylglycerophosphate synthase

**pH**, potential of hydrogen

**PI**, phosphatidylinositol

**PlsC**, lysophosphatidic acid acyltransferase

**PlsX**, phosphate acyltransferase

**PlsY**, glycerol‐3‐phosphate acyltransferase

**POPC**, 1‐Palmitoyl‐2‐oleoyl‐sn‐glycero‐3‐phosphocholine

**POPE**, 1‐palmitoyl‐2‐oleoyl‐sn‐glycero‐3‐phosphoethanolamine

**POPG**, 1‐palmitoyl‐2‐oleoyl‐sn‐glycero‐3‐phosphatidylglycerol

**PPL**, photoredox lipid ligation

**PS**, phosphatidylserine

**PssA**, phosphatidylserine synthase

**PURE**, Protein synthesis Using Recombinant Elements

**PX**, diacylglycerol phosphate with variable headgroup X

**PY**, diacylglycerol with variable headgroup Y

**RNA**, ribonucleic acid

**SecA**, Protein translocase subunit SecA

**SecYEG**, bacterial protein‐conducting translocon

**SepF**, cell division protein SepF

**SMP**, small membrane protein

**SP6**, regulatory promoter for recombinant protein expression

**T7**, regulatory promoter for recombinant protein expression

**WT**, wild type

Over the last two decades, synthetic cells have come to play a central role in the field of synthetic biology, driving significant research efforts and advancements. Indeed, there has been a marked increase in publications on synthetic cells, often also referred to as minimal cells, protocells, artificial cells, or other related terms. This diversity in terminology is accompanied by a wide range of interpretations of how synthetic cells are defined. Descriptions span from simple compartments encapsulating biomimetic molecules to engineered, more complex self‐replicating entities that are considered alive [1, 2, 3]. Recently, leading scientists in the field have engaged in extensive discussions to shed light on the definitions of synthetic cells and their societal impact [4, 5]. Ultimately, the choice of terminology and definition is largely shaped by the research context, which can be broadly categorized into applied and fundamental goals. The applied category emphasizes the practical use of synthetic or artificial cells in fields such as biomedicine, healthcare, industrial biotechnology, nanotechnology, energy, and sustainability [6, 7, 8]. Applications include their use as drug delivery vehicles, biosensors, production hosts, energy generators, etc. In contrast, fundamental research focuses on understanding the principles of life. This includes unraveling the origin of life (astrobiology, prebiotic chemistry, evolutionary biology, etc.), but also general systems biology, as well as efforts toward engineering synthetic or minimal cells that are alive [9, 10, 11, 12, 13].

This Review concentrates on the latter synthetic minimal cells, here defined as out‐of‐equilibrium, self‐replicating units enclosed by a boundary layer and equipped with a basic genetic system and metabolism. The creation of such mimics of living cells has recently garnered significant attention. Numerous initiatives and communities, such as BaSyC (https://www.basyc.nl/about‐basyc/), MaxSynBio (https://www.maxsynbio.mpg.de/home) [14], EVOLF (https://www.evolf.life/), Build‐a‐Cell (https://www.buildacell.org/) [15], FabriCELL (https://www.imperial.ac.uk/a‐z‐research/fabricell/), and SynCellEU (https://syntheticcell.eu/), highlight the growing importance of this field. These initiatives are aimed at unraveling the basic principles of cellular life as well as exploring their future applicability, for example as possible prototypes for next‐generation cell model systems.

In general, two main approaches are being implemented to generate such a synthetic minimal cell: top‐down and bottom‐up. Following the top‐down approach, a cell is stripped down, thereby removing all “redundant” components. This approach focuses on defining minimal genetic and functional requirements necessary for life, thereby reducing the cell's metabolic load, while maintaining viability [13, 16]. However, this strategy is challenged by the complex interplay between genes and function, frequently leading to unintended consequences, such as abnormal phenotypes and/or slower replication [17, 18, 19, 20]. Despite extensive efforts and major advances, current versions of minimal cells contain a great number of essential components, of which the functions are unknown, indicating the gap in our current understanding of the simplest forms of life.

Alternatively, the bottom‐up approach can be used. The idea behind this strategy is to start off with non‐living building blocks that, upon assembly, give rise to a living synthetic entity [21]. This approach has the major advantage that no exact copy of a living entity has to be built, but a novel design can be pursued. In theory, it allows researchers to mix and match a wide variety of existing, but also novel, synthetic sub‐structures, in which natural, but also unnatural/chemical, building blocks can be used. Although such a wide variety of opportunities sounds promising, attempts in the last decade have shown that the bottom‐up engineering of synthetic cells is extremely complex and challenging, requiring joined efforts. Current initiatives are based on the design of cellular sub‐modules (e.g., energy production, genetic code replication, compartment division, etc.), which should then be combined to result in a living cell. Several practical issues, for example, compartment encapsulation efficiency, the sustainability of designed systems, etc., still need to be tackled. Moreover, enabling compatibility and synergy between the designed sub‐systems/modules is of the utmost importance. In this context, perhaps the biggest challenge is formed by synthetic cell division. Not only should the compartment barrier divide without leaking, but the interior lumen also needs to be uniformly distributed over the two daughter cells, which requires precise coordination and timing. In this process, the cell membrane plays an important role.

## Synthetic cell membrane functionality

**Fig. 1:**
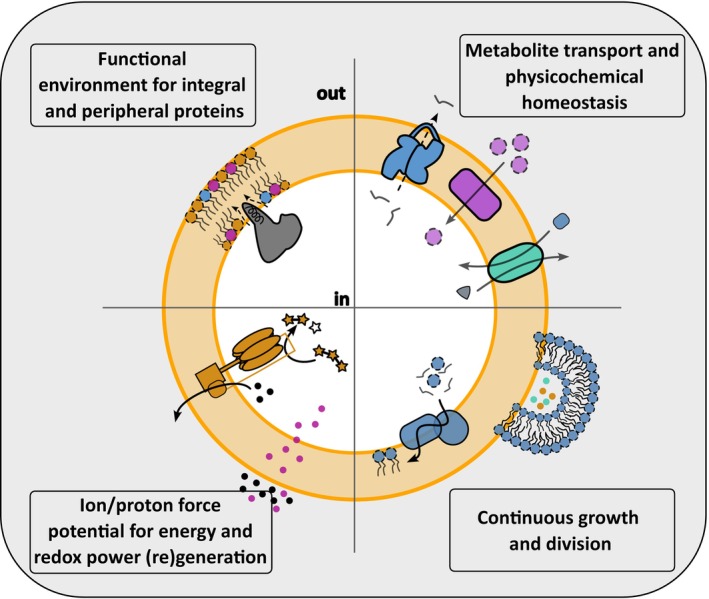
Schematic representation of key functions required for a synthetic minimal cell membrane. Integral and peripheral membrane proteins require a functional membrane environment that supports their structural integrity and enzymatic activity. The membrane serves as a key anchoring platform for the import of essential metabolites and the export of cellular products—both critical for maintaining physicochemical homeostasis. Among the fundamental characteristics of life are growth and division, processes that are tightly linked to lipid biosynthesis and membrane expansion. These functions cannot be sustained without continuous energy generation and regeneration, which in autonomous systems is typically driven by membrane‐associated ion or proton gradients. Created with Inkscape (version 1.2.1).

Among all domains of life, the boundaries of cells are made of a lipid bilayer, also referred to as a membrane. The bilayer functions as a hydrophobic barrier, thereby separating the cell lumen (interior) from the environment (exterior). As a result, the bilayer encompasses a confined reaction space in which molecules can accumulate. In general, this is a key prerequisite that drives enzymatic reactions and is therefore a critical feature of a synthetic cell. As the reaction space is small, and therefore the number of molecules that can be reconstituted is limited, it will not take long until the reactions are complete and an equilibrium is reached. Therefore, a constant supply of new molecules from the environment into the cell's lumen is needed, which requires the passage of the lipid bilayer. In this process, membrane proteins play a specific role, driving the active/passive transport of a wide variety of molecules, such as nutrients and waste products, across the membrane, thereby ensuring an out‐of‐equilibrium system (Fig. 1). The lipid bilayer plays a crucial part in this process, not only forming a suitable matrix for the membrane proteins but also playing an active role in the insertion, structure, function, and mobility of membrane proteins [22, 23, 24].

Besides solute transport, membrane proteins are also involved in metabolic regulation, environmental adaptation, signal transduction across the membrane, etc. Although signaling and adaptation to the environment is imperative for natural cell survival, it might be of lesser importance for the engineering of a synthetic cell, where the environmental conditions can be tuned as the researcher sees fit [25, 26]. Finally, cellular membranes serve as a site for energy conservation, as they enable the build‐up of an ion/proton energy potential across the membrane. For example, above their survival temperature, cells show an increased permeability toward ions, illustrating the importance of the hydrophobic aspect of the bilayer [27]. Moreover, depending on the environmental conditions, the presence of specific lipids is essential to sustain the ion gradient. This is exemplified by the membranes of the archaeal extremophile *Sulfolobus acidocaldarius*, in which the lipid head group, calditol, is essential for growth at extremely low pH [28]. Besides that, the correct insertion of some membrane proteins is dependent on an ion gradient. It is noteworthy that, in eukaryotes, the membranes of the intracellular mitochondria fulfill the role of energy conservation. Such an approach is also considered in the design for a synthetic cell, as it would result in the engineering of simpler subsystems, but at the same time is also considered a major disadvantage in the already complex process of cell division [29].

## Synergy between lipid synthesis and membrane growth

In nature, a huge diversity of different cells exists. To accommodate all the different functionalities, there is a wide variety of membrane compositions, in which the lipid content also differs. This is exemplified by the sheer amount of different lipid (>49 000 species indexed in the LIPID MAPS database), which can vary depending on the classification method used [30]. It should be noted that many of these lipids are only needed under specific conditions or are supporting processes that are not essential for cell viability. Due to this enormous diversity, a wide range of different synthesis routes exists. However, there is a general structure underlying the synthesis of a bilayer‐forming amphipathic lipids (phospholipids, glycolipids, sphingolipids, etc.), which is conserved among the three domains of life (Archaea, Bacteria, and Eukarya). As this has already been extensively described elsewhere [31, 32, 33], we will not go into the details of lipid synthesis, but instead focus on its role in membrane expansion.

## Lipid tail synthesis

**Fig. 2:**
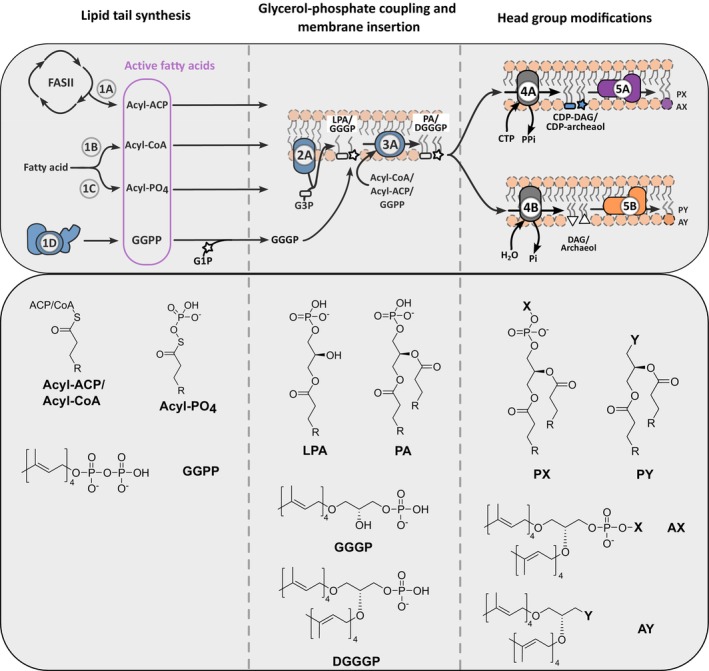
General schematic of (phospho‐)lipid biosynthesis pathways representing all domains of life, divided into 3 sections: Lipid tail synthesis (1A–D), lipid backbone coupling (2A and 3A), and headgroup modification (4A–B and 5A–B). In the first stage, short water‐soluble carbon chains are assembled through multiple rounds of elongation—primarily mediated by the FASII enzyme system in Bacteria and Eukarya (1A), or by the mevalonate pathway in Archaea (1B). Alternatively, fatty acids (FAs) can be taken up directly from the environment, bypassing the complex *de novo* synthesis process (1B‐C). Subsequently, the acyl chain is coupled to a glycerol‐phosphate backbone to form the first glycerophospholipid precursor, lysophosphatidic acid (LPA) (2A). Addition of a second acyl chain to the backbone yields phosphatidic acid (PA), the central precursor of most phospholipids (3A). Finally, a wide variety of headgroups—such as sugars, amino acids, alcohols, and amines—are attached to the lipid backbone, diversifying the lipidome to suit specific cellular needs (4A–B, 5A–B). Abbreviations: ACP, acyl carrier protein; AX, archaeol phosphate with variable headgroup X; AY, archaeol with variable headgroup Y; CoA, coenzyme A; CDP‐archaeol, cytidine diphosphate archaeol; CDP‐DAG, cytidine diphosphate diacylglycerol; CMP, cytidine monophosphate; CTP, cytidine triphosphate; PX, diacylglycerol phosphate with variable headgroup X; PY, diacylglycerol with variable headgroup Y; DGGGP, digeranylgeranylglyceryl phosphate; GGPP, geranylgeranyl diphosphate; GGGP, geranylgeranylglyceryl phosphate; G1P, glycerol‐1‐phosphate; G3P, glycerol‐3‐phosphate; LPA, lysophosphatidic acid; FASII, multi‐enzyme protein fatty acid synthase; PA, phosphatidic acid. Created with Inkscape (version 1.2.1) and ChemDraw Professional (version 23.0.1.10).

The first step in lipid synthesis is the assembly of the lipid tails (Fig. 2). In Eukarya/Bacteria, they are formed out of fatty acids (FAs), whereas in Archaea, they are made up of isoprenoid chains. Despite these differences, their general mode of synthesis is comparable. Through multiple rounds of elongation, short water‐soluble carbon chains are attached and assembled, a process that involves a variety of different enzymes/complexes (Fig. 2_1A) [34]. Alternatively, FAs from the environment can be taken up directly, thereby avoiding the complex synthesis process (Fig. 2_1B,C). Depending on the organism, additional differences in the lipid tail architecture, such as length, saturation, branching, and oxidation, are observed. The variety in lipid tails enables cells to live under a wide variety of conditions but, in a controlled laboratory environment, many of the additional modifications are not essential for cell survival. As the resulting lipid tail is hydrophobic, it is coupled on one end to a carrier group (e.g., acyl carrier protein, coenzyme A, pyrophosphate), which gives the molecule an amphipathic nature/character (Fig. 2). As the other end of the lipid is still hydrophobic, the molecule is poorly compatible with the hydrophilic environment of the cell lumen and needs either proteins, such as FA– or acyl‐CoA–binding proteins, and/or a membrane‐like environment to shield the hydrophobic lipid tail [35, 36].

## Backbone coupling

The lipid‐tail‐carrier group complex serves as the substrate in the next step of lipid synthesis, in which two lipid tails are coupled to a lipid backbone. The two most common backbones are the glycerol‐phosphate (glycerol‐P) backbone (Fig. 2_2A), forming the base for glycerolipids, or L‐serine in the case of sphingolipids. Glycerol‐P coupling is a process that is mediated by two types of membrane‐associated proteins: glycerol‐P acyltransferase (GPAT) and lysophosphatidic acid acyltransferase (LPAAT) [37, 38]. GPAT type enzymes catalyze the coupling of the first lipid tail to the glycerol‐P backbone, resulting in a lysophosphatidic acid (LPA/GGGP; Fig. 2). In turn, LPAAT type enzymes then utilize the formed LPA to attach an additional lipid tail, resulting in the phospholipid phosphatidic acid (PA/DGGGP; Fig. 2_3A). As LPA and PA are located in the membrane, their synthesis automatically also results in membrane expansion. Although these enzymes were identified decades ago, the dual mechanism of lipid synthesis and membrane insertion at the molecular level is still not fully resolved. Specifically, the precise interactions of GPAT and LPAAT with the lipid membrane upon LPA/PA synthesis, as well as the docking of the lipid‐tail‐carrying group substrate, remain unclear. Sphingolipid synthesis involves a similar process, but with different enzymes. Instead of GPAT and LPAAT, the enzymes serine acyltransferase and ceramide synthase (CerS) mediate the coupling of the first and second lipid tail, respectively [39, 40, 41].

## Headgroup attachment

As a final step in lipid synthesis, a headgroup is attached (Fig. 2_4A,B). A wide variety of different molecules can serve as headgroups (e.g., sugars, amino acids, alcohols, amines, etc.), and, as a consequence, a wide variety of different enzymes/enzymatic pathways are involved in lipid headgroup synthesis and attachment (Fig. 2_5A,B) [42]. This process does not further involve membrane expansion, but does influence the specific functionality of the membranes. The wide variety of different headgroups drastically diversifies the lipid landscape, which can impact the functionality of the embedded proteins, as well as energy conservation. Among the lipid classes, anionic phospholipids—such as phosphatidic acid (PA), phosphatidylglycerol (PG), cardiolipin (CL), phosphatidylserine (PS) and phosphatidylinositol (PI)—carry a net negative charge and contribute to a range of essential functions. These include mediating electrostatic interactions with membrane‐associated proteins [43, 44, 45], influencing membrane curvature and dynamics [46, 47], and contributing to membrane potential and the regulation of ionic gradients [48, 49, 50].

In contrast, zwitterionic phospholipids, such as phosphatidylethanolamine (PE) and phosphatidylcholine (PC), exhibit a net neutral charge and are proposed to function as lipochaperones that support proper folding and topology of membrane proteins [51, 52]. Additionally, both anionic and zwitterionic lipids play critical roles in lipid packing and the lateral organization of membrane domains, which are crucial for maintaining membrane structure and function.

These electrostatic and structural characteristics represent only part of the broader physicochemical properties of phospholipids, as they play a critical role in determining the structure and dynamics of biological membranes. The shape of a lipid molecule, cylindrical or cone‐like, depends on the relative size of its head group compared to its hydrophobic tail(s). This geometry influences the intrinsic curvature of the membrane [47]. Cylindrical, bilayer‐prone lipids, such as PC, tend to support flat membrane structures and exhibit minimal curvature‐inducing properties. In contrast, cone‐shaped, non‐bilayer‐prone lipids, such as diacylglycerol (DAG) and lysophosphatidic acid LPA, can induce negative and positive curvature, respectively [46, 47].

In lipid mixtures, non‐bilayer‐prone lipids introduce curvature stress into the membrane and often accumulate in regions with high curvature, such as the cell poles and the division site. These localized domains can support the function and localization of curvature‐sensitive membrane proteins [53]. It is important to note that membrane curvature is not solely governed by lipid composition. Other factors—including curvature‐inducing proteins, ion concentrations, temperature, and pH—also contribute significantly to membrane morphology and dynamics [54, 55, 56]. Despite the wide variety in lipid headgroups, often a minimal subset of headgroups is sufficient to support the basic functionality of the cell [57, 58].

Altogether, the above clearly demonstrates that the construction of a synthetic entity that resembles living cells should include a boundary consisting of lipids that can: (1) fulfill the barrier function, (2) support membrane protein activity, and (3) maintain an ion gradient for energy conservation, thereby illustrating the versatility and complexity of membranes (Fig. 1). As phospholipids play a crucial role in these processes, they should be part of any approach toward the construction of a synthetic cellular boundary layer. Moreover, constructing a self‐replicating synthetic cell requires membrane expansion, a process that is intertwined with phospholipid synthesis. In the following sections, we highlight recent advances by following the top‐down approach, providing novel insights into the minimal requirements of a synthetic cellular membrane. Furthermore, we report on the state‐of‐the‐art in bottom‐up engineering of synthetic cellular membranes, including chemical/artificial equivalents of phospholipids.

## Top‐down approach: Toward minimal membranes

In all life, a substantial amount of the genome is dedicated to membrane‐associated or ‐integrated proteins. Estimates of 20–30% are commonly reported, initially derived from membrane protein prediction studies conducted between the late 1990s and early 2020s using the genome‐wide sequence data available at the time, and later confirmed [59, 60, 61]. With recent advances in both metadata collection and membrane protein annotation, more specific numbers can be provided. By screening the genome of 200 prokaryotes, an estimate of 10–13% of the entire genome was found to encode membrane transport proteins [62]. Moreover, a recent study discovered a new set of so‐called small membrane proteins (SMPs) that were not designated in genome annotations before, due to their small open reading frames [63]. To support all these membrane proteins, a suitable lipidome is required, containing a variety of different headgroups, as well as lipid tails (length, saturation, oxidation, etc.). Following the top‐down approach, detailed insights into the essential requirements and functions of a synthetic cellular membrane can be obtained. Moreover, it serves as a tool to determine the minimal lipid composition necessary for maintaining fundamental membrane properties. Processes that ensure mechanical membrane stability, selective nutrient exchange, and metabolic regulation are essential for maintaining an out‐of‐equilibrium cell state [64]. In contrast, functions such as intercellular communication, endocytosis, exocytosis, environmental responses, and adaptation can be considered non‐essential under defined and controlled conditions [19, 20, 65].

Eukaryotic cells are the most complex among the three domains of life. Approaches toward genome minimalization do exist, for example in *Saccharomyces cerevisiae*, which is widely used in industry and research. By developing several synthetic chromosomes, the 12 megabase pair (Mbp) genome of *S. cerevisiae* was reduced by 8%, although small impairments in organismal fitness are already evident, and debugging remains highly complex [66]. Moreover, the convoluted membrane interactions and organelle dynamics of eukaryotic templates continue to hinder the realization of minimal cell design. Prokaryotes, on the other hand, have less genomic material and lower interaction complexity. For example, the model organism gram‐negative bacterium *Escherichia coli* has a 4.6 Mbp genome [67]. Genome minimization in *E. coli* has resulted in a modest reduction of approximately 8.1–15.3% [68]. Focusing on its phospholipids, only three main species are present: the anionic PG, the zwitterionic PE, and CL. Due to its unique shape, CL preferentially localizes to membrane curvature regions and is suggested to play a role in membrane stability and cell division [69]. However, in a *clsABC‐*null mutant lacking CL, no significant phenotypic changes were observed, indicating that CL is not essential under standard growth conditions and that increased levels of PG may compensate for its function [58, 70]. In contrast, deletion of *pssA*, which encodes an enzyme essential for PE biosynthesis, results in a complete loss of PE. This leads to a disruption in the coordination between cell growth and division, producing a filamentous cell morphology [71]. Additionally, the absence of PE impairs energy‐dependent uphill transport processes, including the uptake of amino acids and sugars [72]. It has been hypothesized that certain lipids, such as PE, may assist membrane‐associated proteins in attaining their correct structural organization in a chaperone‐like fashion. The final topological architecture of these proteins is thought to be influenced by the specific lipid environment of the organism [51, 52]. Anionic phospholipids, such as PG and CL, are believed to play crucial roles in stabilizing membrane proteins through charge‐dependent interactions, thereby supporting their functional integration into the membrane [73, 74, 75]. Interestingly, a *pgsA‐*null mutant, which is deficient in PG synthesis, remains viable and exhibits growth comparable to the wild type [76]. This phenotype may be explained by compensatory accumulation of PA, an acidic precursor of phospholipids.

Although its phospholipid composition is rather simple, *E. coli* possesses a double‐membrane system, consisting of an inner plasma membrane and an outer lipopolysaccharide (LPS)‐rich membrane, which adds an additional layer of structural and experimental complexity, especially when it comes to boundary layer growth and division. Nevertheless, the organism remains a powerful model for investigating membrane–protein interactions and characterizing minimal lipidomes.

## mini*Bacillus* strain PG10

The construction of a minimal membrane for a synthetic cell should begin with naturally occurring membranes of lesser complexity, rather than highly complex ones. Consequently, Gram‐positive organisms, such as *Bacillus subtilis*, serve as preferred models for lipidome minimization. Although *B. subtilis* contains a peptidoglycan layer on top of its lipid membrane, the cell wall is not essential for survival [77]. Hence, the responsible genes can be neglected in the context of a synthetic cell. *B. subtilis* 168 is a widely used laboratory strain, while mini*Bacillus* PG10 is a genome‐reduced derivative, with a 36% reduction compared to *B. subtilis* 168. Despite exhibiting a defect in the final stage of cell division, PG10 is suggested as a promising host for the production of heterologous proteins and peptides [78, 79]. Various genome minimization efforts have targeted membrane proteins associated with adaptivity, including those involved in biofilm formation, motility, and stress adaptation [20]. Despite these deletions, mini*Bacillus* PG10 exhibits robust growth in complex media such as lysogeny broth (LB), albeit with a slight reduction in growth rate and pronounced phenotypic abnormalities that include the membrane. Specifically, PG10 cells form long, undivided filaments and exhibit clumping, indicating a defect in the final stages of cell division [80]. Initial studies on the membrane composition of mini*Bacillus* PG10 suggest a similar phospholipid homeostasis to its parental strain, *B. subtilis* 168. The membrane primarily consists of PE, PG, glycolipids, lysyl‐phosphatidylglycerol (LPG), and CL [81, 82]. Its membrane is predominantly anionic due to the high PG content, but charge gradients can be modulated by the variable presence of the positively charged LPG. Mini*Bacillus* PG10 contains all the above‐stated lipid headgroup species. Moreover, it retains functional membrane microdomains enriched in flotillin, CL, and prenol lipids, indicating that they play an essential role in promoting interaction between particular proteins and supporting their activity [79]. In particular, CL has been identified as a key interaction partner of the membrane fission protein FisB during spore formation [83]. Furthermore, CL contributes to the stability of the b_6_c:caa_3_ respiratory supercomplex in *B. subtilis* membranes [84]. Finally, acidic phospholipids, such as PG, are crucial for the proper membrane localization and function of protein translocase subunit SecA, which is involved in protein translocation across the membrane [85]. Despite these insights, a systematic approach investigating minimal lipidome complexity is still required to better understand the essential roles and interdependencies of specific lipid headgroups in maintaining membrane functionality.

Focusing on lipid tails, a decrease in the relative abundance of FAs is present in the membrane of PG10 compared to *B. subtilis* 168 [79]. Interestingly, a different study on *B. subtilis* included a feed with exogenous FAs, in which it became evident that only a minimal amount of lipid tails is required for cellular growth. By inhibiting endogenous FA synthesis with cerulenin, cell growth could be rescued upon supplying only two FAs: the high‐melting C_16_ and low‐melting anteiso C_15_ FAs [82]. These two FAs give rise to four different acyl chain combinations for the glycerol‐P lipid backbone, which is apparently sufficient to maintain the biophysical properties of the membrane (fluidity and packing). However, the reduced FA pool is compensated by an adjusted lipid headgroup distribution. The PG content is increased, whereas PE levels decrease, which in fact is suggested to enhance membrane fluidity [82].

Altogether, these findings mark important steps toward understanding membrane minimization and reduction of lipid content. On the other hand, mini*Bacillus* PG10 remains far from a truly minimal organism, retaining approximately 2700 genes, while exhibiting compromised fitness, particularly in cell division [20].

## JCVI‐syn3.0

**Fig. 3:**
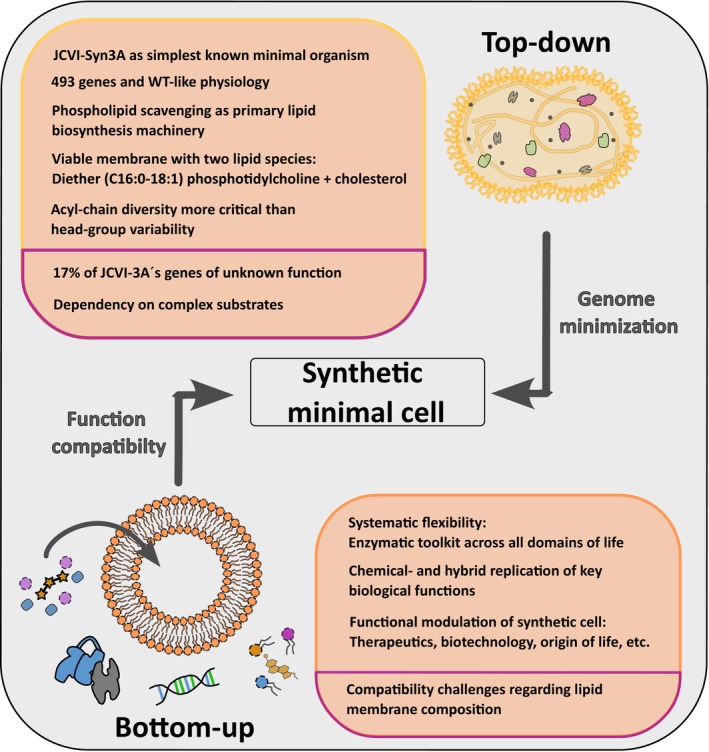
Schematic depiction of the (dis)advantages of bottom‐up and top‐down approaches in minimal membrane system research. In the case of JCVI‐syn3A, the top‐down approach resulted in a successful genome reduction to 493 genes, while maintaining wild‐type‐like physiology. Moreover, its membrane is composed almost exclusively of two lipid species: diether phosphatidylcholine (C16:0–18:1) and cholesterol. At the same time, approximately 17% of JCVI‐syn3A's genome still consists of genes with unknown essential or quasi‐essential functions, and the organism remains highly dependent on the import of complex substrates. In contrast, the bottom‐up approach offers researchers systematic flexibility, allowing them to draw inspiration from and combine enzymatic subsets from all domains of life. Additionally, hybrid and chemical systems may reduce the complexity of synthetic cells by mimicking controllable biological functions. However, these systems face significant challenges, particularly in achieving lipid–protein compatibility, which is essential for providing a functional membrane environment that supports protein activity and stability. Created with Inkscape (version 1.2.1).

Perhaps the most prominent example of the top‐down approach is JCVI‐syn3.0, developed in the lab of John Craig Venter. It was derived from *Mycoplasma mycoides*, a simple bacterial pathogen with a naturally small genome of only 1079 kbp and a single cell membrane, making it an ideal candidate for genome minimization [86, 87]. The JCVI‐syn3.0 chromosome is divided into eight modules (seven native and one synthetic), each individually minimized using transposon mutagenesis. This resulted in a 483 kbp JCVI‐Syn3.0 genome containing only 438 protein‐coding and 35 RNA‐coding (semi‐)essential genes [18]. Notably, 18% of its genome is specifically dedicated to cell‐membrane‐related functions (membrane transporters, lipoproteins, and efflux systems) [87, 88]. Since *Mycoplasma* lacks the biosynthetic pathways for essential metabolites, it instead relies on the uptake of complex nutrients from its environment, which requires crossing the membrane. Additionally, 17% of the JCVI‐syn3.0 genome is allocated to cytosolic processes, including lipid salvage and biogenesis [18]. Another 17% consists of genes with no assigned functional category, although they are likely involved in gene expression, genome maintenance, cytosolic metabolism, and membrane structure and function. Given these distributions, we estimate that approximately 20% of JCVI‐syn3.0 genes are involved in membrane‐associated processes. Although this reduction of the genome is a success, it comes at a cost. JCVI‐syn3.0 exhibits major growth limitations and morphological differences compared to its wild type. It has an increased doubling time, from 60 to 180 min, and instead of forming nonadherent, predominantly single cells with a diameter of 400 nm, it develops into long, segmented filaments accompanied by large vesicular structures [18, 86]. This strongly indicates impairments in the regulation of growth and division, as well as potential defects in intercellular communication, both of which are essential for proper cellular function. Indeed, after reinstating 19 genes, including the cell‐division‐supporting proteins FtsZ and SepF, the quasi‐minimal JCVI‐syn3A cell was created (Fig. 3, Box 1). This minimal cell is spherical, approximately 400 nm in diameter, and has a reduced doubling time of around 120 min. Although FtsZ and SepF are not essential for life, the significant time increase in the division rate and morphological changes suggest a suboptimal state of the organism [89].

Box 1 **Overview of top‐down challenges in minimal cell engineering, with a focus on lipids and viability.**

To date, JCVI‐syn3A is the simplest known minimal organism with a functional lipidome, primarily composed of just two lipid species: diether phosphatidylcholine and cholesterol. This composition provides valuable insights into the *in vivo* requirements for fundamental cellular membranes. With a single plasma membrane and largely lacking biosynthetic machinery for building block production, JCVI‐syn3A depends on the uptake of complex nutrients from its environment (Fig. 3). This raises a critical question: is complex nutrient uptake or *de novo* building block synthesis more essential for the function and viability of a synthetic cell?

Additionally, approximately 17% of JCVI‐syn3A's genome remains of unknown function, complicating further genome minimization efforts. Large‐scale studies suggest that *Bacillus subtilis* requires 523 and 119 protein‐ and RNA‐coding genes, respectively, for growth under defined laboratory conditions [124, 125]. However, even after extensive genome reduction, Mini*Bacillus* PG10 still contains around 2700 genes.

For both Mini*Bacillus* PG10 and JCVI‐syn3A, genomic minimization resulted in significant phenotypical changes and increased growth rates. Thus, extensive studies and systematic investigations into minimal lipidome complexity are still necessary to refine the design of synthetic cell membranes in the top‐down approach.

Focusing specifically on the lipids, the original *Mycoplasma mycoides* is an exceptional organism as it largely lacks the biosynthetic machinery to produce its own lipids and building blocks, and instead relies on the uptake/scavenging from its host organism and environment (Fig. 3). Remarkably, cholesterol is essential for *M. mycoides* growth, which is rather unusual, as sterols/hopanoids are mostly non‐essential for bacteria [88]. *Mycoplasma mycoides* lacks the synthesis pathway of cholesterol and instead incorporates it through physical adsorption, a process that is dependent on phospholipid composition, temperature, and membrane fluidity [88]. Specific receptor proteins located on the *Mycoplasma* surface increase the efficiency of this process by mediating contacts with PC‐cholesterol vesicles or serum lipoproteins [88]. Besides cholesterol, other sterols, phospholipids, and free FAs that are present in the environment are taken up, where they can be modified or scavenged [88, 90]. For example, *M. mycoides* can modify the acyl chain composition of its membrane lipids through the hydrolysis of exogenous phospholipids by phospholipases. However, this process is strictly constrained by the lipid tail configurations available in the surrounding environment. Besides altering the acyl chain configuration, the scavenged lipids can be utilized to actively synthesize PG, which can be further modified into CL.

In short, the modification/scavenging of environmental lipids, instead of using a more complex lipid biosynthesis machinery, results in a simple system, with a small lipidome. These properties were leveraged to study the essential lipid composition required for minimal life [91]. By minimizing the lipid feed to a combined diet of oleic acid (18:1), palmitic acid (16:0), and cholesterol, a viable *M. mycoides* was obtained, consisting of nine lipid species (two DAGs, one PA, two PGs, three CLs and cholesterol; Table 1). The two free FAs could also be substituted by a single phospholipid, 16:0/18:1 phosphatidylcholine (POPC), which resulted in a lipidome consisting of 18 lipid species: mostly PC and cholesterol, with small amounts of CL, PG, and DAG from POPC scavenging (Table 1). Unexpectedly, minor amounts of PC species with a 17:1 lipid tail and an 18:2 lipid tail were detected. Whether these are enzymatically processed from POPC, or simply contaminations of the POPC stock, remains unclear. Next, the ester‐coupled lipid tails of POPC were exchanged by an ether‐coupled 16:0 and 18:1 acyl‐chain, thereby bypassing the lipase activity and subsequently lipid scavenging. Both wild‐type *M. mycoides* as well as JCVI‐syn3A could survive on this diet, which resulted in a functional minimal lipidome composed of only two lipid species, diether 16:0–18:1 PC (D. PC) and cholesterol (Fig. 3; Table 1). To put these results into perspective, the smallest experimentally achieved lipidome in a living organism consists of 27 lipid species, excluding unanalyzed LPSs [92]. Although these cells are viable, the lipidome restrictions come at a cost. A two‐fold increase in growth rates and a higher likelihood of internal membrane invaginations were observed, suggesting compromised spatiotemporal regulation of cell size and shape [91]. Interestingly, although phospholipid headgroup diversity does not significantly affect the growth rate of *Mycoplasma*, variations in acyl chain composition can rescue it, highlighting the importance of proper acyl chain configuration for optimal membrane properties and lipid–protein interactions.

**Table 1:** Membrane lipid composition of *Mycoplasma mycoides* and JCVI‐syn3A in diet conditions. Modified from supplementary data of [91].

Feature	*Mycoplasma mycoides* oleic and palmitic acid + cholesterol diet (%)	*Mycoplasma mycoides* POPC + cholesterol diet (%)	*Mycoplasma mycoides* diether PC + cholesterol diet (%)	JCVI‐syn3A diether PC + cholesterol diet (%)
DAG [16:0 | 16:0]	1.03036253	0.314721603		
DAG [16:0 | 18:0]		0.508779196		
DAG [16:0 | 18:1]	0.758715397	1.314809255		
DAG [18:0 | 18:0]		1.349799346		
PA [16:0 | 18:0]	0.287965122			
PC [16:0 | 16:0]		1.595259107		
PC [15:0 | 18:1]		0.6609825		
PC [16:0 | 17:1]		0.293845499		
PC [16:0 | 18:1]		26.52832744		
PC [18:0 | 18:0]		0.692048118		
PC [18:0 | 18:1]		0.386160177		
PC [18:0 | 18:2]		0.685676154		
PC [diether 34:1]			47.7250431	48.38691481
PG [16:0 | 16:0]	9.95861932			
PG [16:0 | 18:0]		0.35876648		
PG [16:0 | 18:1]	9.738556979	0.423468667		
PG [18:0 | 18:0]		2.624122047		
CL [66:1]	8.185994622			
CL [68:1]		0.573757278		
CL [68:2]	10.55451109			
CL [70:1]		0.420281351		
CL [70:3]	0.229523563			
CL [72:1]		0.395031207		
Cholesterol	58.46788095	59.9281599	52.18263407	50.9562864
Other	0.787870422	0.94600468	0.092322833	0.656798795

## Bottom‐up approach: Toward synthetic membranes

The top‐down approach provides insights on essential membrane components and minimal lipidomes in already existing organisms, which can be utilized in the bottom‐up construction of a synthetic cell. At the same time, the bottom‐up approach is not limited to a single organism, but can implement a variety of enzymatic pathways originating from different organisms (Fig. 3). Although this opens routes for creative and smart design, it also introduces challenges in achieving system cross‐functionality and compatibility at the level of a living organism. Essential functions such as gene expression, genome replication, energy generation, and membrane expansion in a dividing compartment must work together to ensure autonomous, life‐like behavior. In this approach, these functions are built individually, and later need to be integrated and tested in the context of model organism characteristics.

## Membrane protein systems

To date, numerous bottom‐up‐based membrane systems containing peripheral and/or integral membrane protein(s) have been developed in the context of a synthetic/artificial cell. Often, the proteins have been reconstituted into some form of a model membrane, consisting of a specific lipid composition. Hence, a wide variety of different lipid compositions are reported in the literature, differing in lipid headgroup ratios, as well as acyl chain configurations. Consequently, integration of these systems potentially will give rise to compatibility issues.

Table 2 lists several membrane proteins studied in the context of a synthetic cell, focusing on the specific requirements regarding the lipid composition that was reported. Please note that the list comprises only a subset of the vast amount of membrane proteins that have been studied, and therefore serves as an example to illustrate the complexity of compatible integration of these proteins in the context of a synthetic cell. Most of the reported research describes protein activity in different lipid compositions, in which the focus is on lipid headgroups. Some proteins are clearly dependent on a specific lipid headgroup, whereas others show a more general dependency on the charge (anionic, zwitterionic, cationic). Besides the type of lipid headgroup, the concentration can also play a role, which can result in a conflict of interest. This is exemplified by the mitochondrial ADP: ATP antiporter AAC and the ABC transporter OpuA. AAC activity strongly increases in the presence of CL, whereas OpuA activity is inhibited by CL (Table 2) [93]. Another example is the dependency of the translocon SecYEG and the phospholipid‐synthesizing enzyme PlsB on the non‐bilayer zwitterionic PE [94, 95]. Both proteins show enhanced activity in the presence of substantial amounts of PE. By contrast, giant unilamellar vesicle (GUV) formation and stability is impaired in the presence of PE, which was exemplified by the reconstituted Min system (Table 2) [96]. Finally, some eukaryotic proteins, such as the ABC transporter P‐gp [97], benefit from the presence of cholesterol, whereas some prokaryotic proteins are inhibited (Table 2) [98]. Besides protein activity, correct insertion of proteins can also be influenced by lipid composition. This is exemplified by the lactose importer LacY, which requires >50% of PE to obtain its native topology (Table 2) [99]. Similarly, the light‐driven proton pump pR switches its topology depending on the presence of anionic vs. cationic lipids in the membrane (Table 2) [100].

**Table 2:** List of membrane proteins in synthetic membrane systems. Biotinyl CAP PE, 1,2‐dioleoyl‐sn‐glycero‐3‐phosphatidylethanolamine‐N‐(cap biotinyl); CL, cardiolipin; DHPE‐TexasRed, 1,2‐diheptadecanoyl‐sn‐glycero‐3‐phosphatidylethanolamine‐TexasRED; DOPE, 1,2‐dioleoyl‐sn‐glycero‐3‐phosphatidylethanolamine; DOPA, 1,2‐dioleoyl‐sn‐glycero‐3‐phosphate; DOPC, 1,2‐dioleoyl‐sn‐glycero‐3‐phosphatidylcholine; DOPG, 1,2‐dioleoyl‐sn‐glycero‐3‐phosphatidylglycerol; DOPS, 1,2‐dioleoyl‐sn‐glycero‐3‐phosphatidyl‐L‐serine; DOTAP, 1,2‐dioleoyl‐3‐trimethylammonium propane; DPPC, dipalmitoyl phosphatidylcholine; DSPE‐biotin, 1,2‐distearoyl‐sn‐glycero‐3‐phosphatidylethanolamine‐biotin; EYPC, egg yolk phosphatidylcholine; GUV, giant unilamellar vesicle; PA, phosphatidic acid; PC, phosphatidylcholine; PE, phosphatidylethanolamine; PEG2000PE, polyethylene glycol‐2000 phosphatidylethanolamine; PG, phosphatidylglycerol; PI, phosphatidylinositol; POPC, 1‐palmitoyl‐2‐oleoyl‐sn‐glycero‐3‐choline; POPE, 1‐palmitoyl‐2‐oleoyl‐sn‐glycero‐3‐phosphatidylethanolamine; POPG, 1‐palmitoyl‐2‐oleoyl‐sn‐glycero‐3‐phosphatidylglycerol; TOCL, tetraoleoylcardiolipin.

Name, organism, ID (uniprot), reference	Cellular function and relevance	Synthetic membrane system	Requirements for biological activity
Bacteria			
OpuA *Lactococcus lactis* A0A0V8ETW8 [126]	ABC transporter; glycine betaine uptake Osmoregulatory protein during stress	DOPE:DOPG:DOPC (50:38:12%) Different compositions have been tested by varying DOPG and DOPE contents from 0% to 50%	DOPG content must be >18% (optimally >25%) CL has inhibitory effect on activity Increasing DOPE content leads to higher activity
pR SAR (uncultured marine gamma proteobacterium) (not classified) [100]	Light‐driven proton pump; harvest light energy Engineering of light‐controlled systems	Neutral: 100% DOPC Negatively charged: POPC:POPG (80:20%) Positively charged: DOPC:DOTAP (80:20%)	C‐exposed orientation for positive liposomes, inward pumping of protons N‐exposed orientation for negative liposomes, outward pumping of protons
CcO *Rhodobacter sphaeroides* P33517 [127]	Electron transfer/ proton pump; Relevant for oxygen utilization, engineering of energy systems	Native lipid extracts: Soybean PC (94.6% PC)*^1^, *E. coli* polar lipid extract: (66.7:23.1:9.8% PE:PG:CL) Synthetic lipid mixtures: 1. PE:PC (66.7:33.3%) 2. PE:PG:PC (66.7:23.1:9.8%) 3. PE:PG:CL (66.7:23.1:9.8%) *^1^remaining lipids unknown	Reconstitution promoted by anionic lipids (CL and PG) Native lipid extracts lead to more efficient reconstitution than synthetic mixtures (*E. coli* polar lipid extract same as 3. synthetic mixture, amount of reconstituted CcO differs)
LacY *Escherichia coli* P02920 [99]	Symporter, lactose and proton influx Lactose as an energy source	PE content varied from 0% to 70%, PG and CL used to add up 100%	Protein topology changed with increasing PE content: 70% – native topology, 0% – inverted topology, 50% – 2/3 native topology
SecYEG *Escherichia coli* P0AG99 (SecY), P0AGA2 (SecE), P0AG96 (SecG) [95, 107, 128, 129]	Protein translocation/insertion machinery Insertion of newly synthesized membrane proteins in a synthetic cell	PC:PE:PI:PA (24.0:18.6:11.5:4.3%)*^2^ 30% DOPE, 0% – 30% DOPG, 40% – 70% DOPC DOPC:DOPE:anionic lipid (40:30:30%) 30% DOPG, 0% – 70% DOPE *^2^remaining lipids unknown	CL depletion leads to impaired protein translocation 10% PG fully saturates SecA:SecYEG binding, translocation rates increase up to 30% PG SecA:SecYEG translocation dependent on anionic lipids SecYEG mediated protein insertion is dependent on PE content, with an optimum at 60%.
F_o_F_1_ ATPase *Bacillus sp*. *PS3* P09218, P09221, P00845, P09219, P07677, P09222, P07678 [130]	ATP synthase via proton translocation Engineering of energy harvesting systems	PC extract from soybeans +30% chol. POPC:chol.:PEG2000PE (57.5:40:2.5%)	F_o_F_1_ ATPase‐Liposomes as organelles in GUVs 30% cholesterol in the liposomes leads to lower proton leak through the membrane by 1/3
PlsB *E. coli* P0A7A7 [94]	LPA synthesis Component of lipid synthesis machinery; required for growth	DOPC:DOPE:DOPG (33.3:33.3:33.3%)	Activity decreased roughly 40–50% in absence of PE. Activity decreased roughly 50–60% in absence of PG Activity not dependent on PC
Min system *E. coli* MinD: P0AEZ3, MinE: P0A734, MinC: P18196 [96]	Oscillating system; positioning of FtsZ division ring membrane remodeling system	POPC:POPG:Biotin. CAP PE (69:30:1%) POPC:POPG:Biotin. CAP PE:DOPE (69:30:1%)	15% DOPE decreased the number of produced vesicles drastically, but successful reconstitution with 10% DOPE
Eukaryotes			
AAC *Thermothelomyces thermophila* G2QNH0 [93]	Mitochondrial ADP:ATP antiporter Energy metabolism/ATP regeneration systems	DOPE:DOPC:DOPG:TOCL (50:12:35:3%) DOPE:DOPC:DOPG:TOCL (25:47:25:3%)*^4^ *^4^Different lipid composition for OpuA containing vesicles to optimize activity	AAC activity increases with 10% CL, but experiments were done with 3% to ensure compatibility
Cx43 (Connexin‐43) *Rattus norvegicus* P08050 [131]	Formation of gap junctions; allow for ion/small metabolite exchange Regulating cell polarity/migration	DOPC:DPPC or DOPC:DOPG 100:0%, 80:20% and 50:50% Lipid concentration ranged from 1 mm to 30 mm	Integration of Cx43 increased with higher lipid concentration and decreased with rising liposome size and negative charge*^4^ *^4^Influence of the lipid tail desaturation was not mentioned
P‐gp *Mus musculus* (P388/ADR) (not classified) [97]	ABC transporter, functions as efflux pump	POPC 100% POPC/cholesterol 80:20% POPC/cholesterol 60:40%	20% and 40% cholesterol increase the ATPase basal activity by 6 times
Tim23 *Triticum aestivum* (not classified) [132]	Tim23, subunit of the mitochondrial inner membrane transport complex protein import into synthetic cells	POPC:POPE:TOCL (34:32:34%)*^5^ *^5^mimic mitochondrial inner membrane with a lipid composition of POPC:POPE:TOCL (40:40:20%)	High amounts of CL are required for activity

Overall, these examples clearly indicate the difficulties with combining existing *in vitro* membrane systems and illustrate that, in the design and engineering process of a synthetic cell, compatibility with a mutual lipid composition is of the utmost importance. To facilitate this process, the aim of bottom‐up engineering of lipid synthesis and insertion should be a versatile and tunable lipid membrane, such that a wide variety of different membrane systems can be integrated.

## Lipid synthesis approaches

Over the past two to three decades, multiple attempts have been made to synthesize and insert phospholipids into vesicles, thereby mimicking the expansion of a synthetic cellular membrane. Here, we will only focus on recent advances, as the early research has already been extensively described in previous reviews [101, 102, 103].

Although initial simple bottom‐up systems exist out of a limited number of proteins, eventually a synthetic cell will consist of a vast number of different proteins, which require some form of a genetic system for their production. The *in vitro* PURE (Protein synthesis Using Recombinant Elements) system, which contains 32 purified transcription and translation components, has demonstrated the capability to produce active proteins from a genomic template in significant amounts [104]. This system also functions within enclosed liposomes, enabling protein biosynthesis inside a compartment, and thus forming one of the foundational elements for an autonomous synthetic cell in the enzymatic approach. The PURE system was utilized by Eto et al. to establish the early steps in phospholipid biosynthesis, that is, acyl chain synthesis and subsequent attachment to the glycerol‐P lipid backbone [105]. In this process, eight FA‐binding enzymes of the Fab family, along with acyl carrier protein (ACP) and thioesterase 1 (TesA) from *E. coli*, convert the substrates acetyl‐CoA and malonyl‐CoA into acyl‐ACP (Fig. 2). Acyl‐ACP is then modified by the phosphate acyltransferase PlsX to form acyl‐phosphate (acyl‐PO_4_), which serves as a substrate for the GPAT PlsY, leading to the formation of LPA (Fig. 2). A second acyl chain is subsequently attached by LPAAT PlsC, generating PA, the simplest form of a phospholipid, which serves in bacteria as a precursor for phospholipid head group synthesis (Fig. 2). Notably, the PlsX/Y/C system consists of two peripheral and one integral membrane protein. PlsX membrane localization and activity are enhanced by negatively charged lipids, such as PG, whereas PlsC benefits from the zwitterionic PE [94]. To improve PlsX solubility, DnaKJE chaperones from *E. coli* were additionally included. PA synthesis was successfully achieved in GUVs composed of 80 mol% POPC and 20 mol% POPG. However, the maximal concentration of 100 μm was insufficient to induce observable morphological changes in the membrane vesicles. Malonyl‐CoA synthesis, potentially regulated by acyl‐ACP intermediates, may act as a rate‐limiting step in PA synthesis. Moreover, the system lacks an integral membrane protein insertion system, which could be beneficial for PlsY activity. Finally, mechanisms for substrate import and waste export are missing, potentially leading to the accumulation of intermediates.

In a similar approach, the upgraded PURE*frex*2.0 transcription and translation system (using orthogonal T7 and SP6 promoters for controlled expression) was used to synthesize phospholipids in liposomes, this time focusing on headgroup integration (Fig. 2) [106]. The researchers were able to successfully synthesize six different phospholipid end products: 1,2‐dioleoyl‐sn‐glycero‐3‐phosphoethanolamine (DOPE), 1,2‐dioleoyl‐sn‐glycero‐3‐phosphoglycerol (DOPG), 1,2‐dipalmitoyl‐sn‐glycero‐3‐phosphoethanolamine (DPPE), 1,2‐dipalmitoyl‐sn‐glycero‐3‐phosphoglycerol (DPPG), 1‐palmitoyl‐2‐oleoyl‐sn‐glycero‐3‐phosphoethanolamine (POPE), and 1‐palmitoyl‐2‐oleoyl‐sn‐glycero‐3‐phosphoglycerol (POPG). However, from the perspective of cell growth, the amount of lipid produced inside the GUVs (DOPC/DOPE/DOPG/CL/DSPE‐PEG‐biotin; 50 mol%/36 mol%/12 mol%/2 mol%/1 mass%) was relatively low, leading to no observable membrane expansion. Possibly, the poor solubility of initial substrate acyl‐CoA limits substrate availability. Moreover, the accumulation of the phospholipid intermediates LPA, PA, and CDP‐DAG was observed, potentially limiting synthesis rates through feedback inhibition. Especially, the lack of proper insertion of the multi‐membrane spanning proteins CdsA (PA –> CDP‐DAG) and PgsA (CDP‐DAG –>PGP) could be a pivotal bottleneck in this system.

An alternative for the FAS/PlsX/Y system for LPA synthesis is the FadD/PlsB system from *E. coli* [94]. FadD is involved in the β‐oxidation of FAs and can directly utilize FAs, thereby providing a shortcut for the complex synthesis of FAs. FadD and PlsB were purified (instead of produced by PURE), together with six enzymes involved in phospholipid biosynthesis from *E. coli* and *B. subtilis* (PlsC, CdsA, PgsA, PgpA, PssA, and PsD), and reconstituted into pre‐existing liposomes. A feed with FAs and other building blocks resulted in significant conversion of FAs into the phospholipid end products PG and PE, which resulted in substantial membrane expansion. To illustrate this, phospholipid synthesis was coupled to protein translocation by the bacterial translocon, which is dependent on anionic phospholipids. Synthesis and insertion of PG (up to 25% of the total lipid content) into a zwitterionic vesicle activated the embedded translocons, which resulted in translocation of external ProOmpA into the lumen of liposomes [107]. Notably, this inside‐out system synthesizes phospholipids in the outer leaflet of the membrane, thereby surpassing the limitation of substrate availability as previously observed in the lumen of liposomes [106]. Furthermore, minimal accumulation of intermediates was observed, emphasizing the importance of correct folding and insertion of membrane proteins. Ultimately, this system shows that substantial expansion of a vesicle membrane by utilizing the designed enzymatic phospholipid biosynthesis routes is possible. Moreover, it indicates that current *in vitro* transcription/translation coupled approaches in fact can encode a robust phospholipid biosynthesis route, as the same enzymes are used. However, optimizations are needed, which include, among others, continuous feed of substrate, substantial protein production levels, and proper membrane protein insertion.

## Lipid synthesis in an organelles‐like system

As mentioned in chapter “Synthetic cell membrane functionality” the membranes of synthetic cells need to fulfill a wide variety of different functions. Current bottom‐up approaches already show potential bottlenecks when it comes to the lipid membrane compatibility of engineered systems. Another perspective on this is to divide the tasks over several different membranes/organelles. Bailoni et al. followed up on this idea and developed a two‐liposome compartment system that separates lipid (PA) synthesis from energy and lipid building block production, thereby mimicking synthetic organelles *in vitro* [29]. This system integrates nutrient import, cofactor recycling, and lipid building block export, which is segregated from lipid synthesis. In short, the *L*‐arginine pathway (ArcABCD) generates ATP by importing and metabolizing *L*‐arginine into *L*‐ornithine, CO_2_, and NH_4_^+^. *L*‐ornithine is then exported via the ArcD symporter in exchange for *L*‐arginine. Glycerol, which passively diffuses through the phospholipid bilayer, is phosphorylated by GlpK to form the lipid building block glycerol‐3‐phosphate (G3P), which is subsequently exported via the glycerol‐3‐phosphate/phosphate (GlpT) antiporter. As a result, one set of the liposomes receive *L*‐arginine, phosphate (Pi), and glycerol—either passively or via the ArcD and GlpT transporters—while releasing *L*‐ornithine, G3P, NH_4_^+^, and CO_2_ into the environment. The counterpart liposomal set is equipped with the phospholipid biosynthesis enzymes PlsB and PlsC, and, in combination with FadD, CoA, ATP, FAs and the synthesized and excreted G3P, are able to synthesize PA on the outer surface of the liposome. Although this setup provides a proof‐of‐principle for a donor–acceptor relationship, the diffusion of G3P seems to act as a limiting step in PA synthesis, which results in a theoretical estimated doubling time of no less than 10 days. Practically, not only membrane growth, but also division, form enormous challenges, as the interaction network across multiple sub‐compartments/organelles significantly increase systems complexity. Nevertheless, multicompartment synthetic cells benefit from an improved surface‐to‐volume ratio provided by organelle‐like networks, which would in turn enhance metabolite exchange and membrane‐associated processes [108]. In theory, the complexity of synthetic cells could be distributed across a network of specialized “expert” cells, each with defined functions, overcoming the limitations of a single‐compartment system. However, the engineering of such complex systems is currently beyond reach.

## Chemical and hybrid phospholipid biosynthesis

One of the major advantages of the bottom‐up approach is the opportunity to drift away from a single organism, and instead implement biological, as well as chemical, alternatives in the engineering design (Fig. 3). For example, in the example above, the *E. coli* phosphatidylserine synthase (PssA) was exchanged with its *B. subtilis* homolog, which exhibited significantly higher enzymatic activity under the given experimental conditions [109]. Similarly, in the *L*‐arginine‐dependent ATP recycling system, the antiporter ArcD was initially sourced from *Lactococcus lactis* but was later substituted with the *Lactobacillus sakei* variant, thereby significantly accelerating the rate‐limiting step of energy recycling within the compartment [29]. An illustrative example of cross‐species biochemical integration involves the fatty acyl adenylate ligase FadD10 from *Mycobacterium tuberculosis*, which catalyzes the formation of fatty acyl adenylates (FAAs) from saturated FAs, Mg^2+^, and ATP [110, 111]. It is noteworthy that these FAAs can undergo non‐enzymatic reactions with primary amines, such as those on chemically functionalized lysolipids, leading to the formation of membrane‐building amidophospholipids in aqueous environments [112]. This characteristic has been utilized in a cell‐free expression system (PURExpress^®^), where the *E. coli* transcription–translation machinery continuously synthesizes FadD10, enabling the *in situ* generation of vesicular structures composed of amidophospholipids [111].

Besides these biology‐based enhancements, other chemical strategies can be implemented for optimization (Fig. 3). Direct aminolysis provides a non‐enzymatic method for constructing amide‐linked phospholipid analogs in aqueous environments. Upon assembly, the water‐soluble building blocks spontaneously self‐assemble into vesicular structures [113]. Additionally, coupling amine‐functionalized lysophospholipids with thioester acyl donors enables the synthesis of native lipids, such as POPC, through imidazole‐promoted esterification, thereby bypassing the need for a complex enzymatic machinery [114]. Both aminolysis and esterification reactions are significantly enhanced by high concentrations of additives such as imidazole or silver ions, offering a controllable strategy for membrane formation and growth. However, the use of non‐physiological reagents and reaction conditions may hinder compatibility with other biological processes, posing challenges for integration into more complex synthetic cell systems.

Furthermore, Cho et al. discovered that cysteine can spontaneously react with two short‐chain thioesters (C8) to form diacyl lipids [115]. These lipids then self‐assemble into compartment systems that are functionally compatible with encapsulated ribozymes. Despite their synthetic accessibility, short‐chain lipids lack sufficient hydrophobic tail length to support robust membrane barrier function. Of note, the lipid head group can be extensively modified through diacylation of N‐terminal cysteine‐containing peptides, highlighting the potential for significant structural diversity and functional versatility.

Another promising approach to chemical lipid synthesis is photoredox lipid ligation (PPL), which involves the reaction between *N*‐hydroxyphthalimide (NHPI) fatty esters and olefin‐modified lysolipids. As a result, natural lipids, such as PCs, sphingolipids, and DAGs can be formed through carbon–carbon bond formation [116]. As the system is light‐induced, it allows in principle for tunable control of compartment self‐replicating processes, such as membrane vesicle formation, membrane growth, budding, and division. Moreover, this system is compatible with living cells and capable of not only phospholipids, but also ceramide and DAGs within living cell membranes [116]. Additional strategies have been described for constructing dividing‐cell‐like systems, including those encapsulating DNA replication machinery [117] and glucose‐oxidase–horseradish‐peroxidase cascade reactions [118] using chemically modified lipid precursors.

Overall, chemical approaches to phospholipid synthesis and modification offer significant advantages for controlled membrane formation and growth, without the complexity of biological systems. However, their application in synthetic cell research is often limited by the use of non‐physiological catalysts and experimental conditions that are incompatible with biological processes.

## Discussion

The development of a synthetic cellular membrane with full functionality is a complex process. The essential cellular tasks a membrane fulfills are numerous and involve a wide variety of membrane proteins, as well as different lipid species for support. Recent developments toward a minimal cell following the top‐down approach have provided novel insights into essential membrane components and minimal lipidomes in living organisms. Focusing on the lipidome, perhaps the most advanced example is JCVI‐syn3A. This minimal life form lacks a biosynthetic machinery for lipid production, instead depending on the uptake and scavenging of complex lipids/building blocks from its environment. By pushing the limits, JCVI‐syn3A maintained a functional membrane composed of more than 99% diether phosphatidylcholine (PC 34:1) and cholesterol (Table 1).

Comparing this with current bottom‐up approaches toward an expanding membrane, most initiatives are based on a biosynthesis machinery for the production and coupled membrane insertion of phospholipids. Although internal lipid synthesis is the most common life form, the machineries are often rather complex, consisting of numerous components. To circumvent this, bottom‐up approaches already have implemented shortcuts, for example, by replacing lipid tail synthesis that starts from simple building blocks with direct FA incorporation derived from an external feed. However, further improvements to reduce complexity should be considered, such as utilization of lipid uptake/scavenging as observed in JCVI‐syn3A, as well as chemical phospholipid analogs. In the search for an expanding synthetic cellular lipid membrane, partial integration and mixing of the above strategies should not be excluded. This is already exemplified by the integration of elements from *E. coli* and *M. tuberculosis* with chemically modified lipid precursors to produce membrane‐forming lipids, which may function as growth metabolites or contribute to compartment formation in synthetic cell systems [111].

Current bottom‐up engineering approaches of a lipid membrane focus mainly on the lipid headgroups. Indeed, particular phospholipid headgroups support specific membrane proteins, exemplifying their importance for proper membrane functioning. As a consequence, the hydrophobic lipid tails are often overlooked. The lipid tail configuration directly affects the biophysical properties of the membrane, that is, bilayer packing/fluidity, which in turn influences the functionality of the embedded membrane proteins. This importance was clearly illustrated by JCVI‐syn3A, in which a feed with either oleic acid (C18:1) or palmitic acid (C16:0) was not viable, showing the importance of a diverse acyl chain configuration for optimal membrane properties and lipid–protein interactions [91].

The other critical membrane constituent is cholesterol, which also has a drastic influence on the biophysical properties of a membrane. Cholesterol is a uniquely versatile lipid capable of modifying the bending rigidity and spontaneous curvature of cell membranes, providing the ability to withstand mechanical stresses [119, 120, 121, 122]. At the same time, the lipid headgroup variety could be reduced to one species only (choline), suggesting that phospholipid headgroup diversity/charge does not significantly affect the growth rate of *Mycoplasma*.

These unexpected findings shed new light on the importance of lipid head groups vs. lipid tails. The negative curvature provided by conical cholesterol, together with the cylindrical D. PC, could be sufficient to modulate membrane fluidity and bending rigidity under defined conditions [123]. Additionally, the neutral surface charge of the zwitterionic PC seems to be sufficient as a functional environment for membrane transporters.

Nevertheless, it is important to put these findings into perspective. Decades of membrane protein research have shown the importance of various lipid headgroups (anionic, zwitterionic, cationic) in a wide variety of cellular processes. This becomes to some extent already apparent for JCVI‐syn3A as well, as the minimal lipidome described above is accompanied by impaired growth and the arise of internal membrane invaginations, indicating compromised spatiotemporal regulation of cell size and shape. Looking at this from a broader perspective, an autonomous synthetic cell requires a segregation machinery for proper partitioning into parent and daughter cells to ensure efficient production of viable progeny. In this process, a tight interplay with the membrane boundary layer is required, in which multiple anionic lipids play an important role.

Overall, numerous interactions between the membrane and its integrated/associated proteins must be carefully considered. For this reason, the primary focus for the bottom‐up engineering of a synthetic cellular membrane should not be on total minimization of the lipid composition. Instead, optimal support of membrane protein functioning is required, such that all essential functions of a membrane boundary layer (growth, division, etc.) are fulfilled. In this process, a wide variety of different membrane proteins derived from various organisms can be considered, as long as compatibility is guaranteed. In particular, combining eukaryotic systems with bacterial systems can be problematic due to the dependency on substantial amounts of sterols (cholesterol, ergosterol, etc.), which greatly impact membrane fluidity and packing. An exception is formed by proteins derived from mitochondria, which contain lower amounts of sterols. That said, mitochondrial membrane proteins often benefit from a high CL content, which can also result in compatibility issues (Table 2). In general, the required ratio between the different lipid headgroup species can drastically vary between proteins from different organisms, thereby also causing potential integration issues during bottom‐up engineering. Furthermore, many bacteria contain substantial amounts of the non‐bilayer‐forming lipid PE, which is a destabilizing lipid for GUVs, which are currently the most used life‐like envelope mimic of a natural cell. To sum up, the bottom‐up approach toward the engineering of a synthetic cellular membrane should be based on a lipid membrane that is simple, yet versatile enough to optimally support membrane proteins, in which compatibility between proteins and lipid membrane is key.

Finally, top‐down research toward minimal cells makes it evident that the minimal requirements for life are still beyond our current understanding. So far, all minimized genomes contain a substantial percentage of unknown genes that are essential for cell viability. For example, in JCVI‐syn3A, approximately 17% of the genome remains of unknown function, likely including a substantial amount of membrane proteins. For the bottom‐up construction of a synthetic cellular membrane, it is therefore important to focus not only on engineering approaches of currently known enzymes/enzymatic pathways, but also on discovering the specific role of the unknown parts that play a role in cell viability.

## Author contributions

SG: Conceptualization (equal); Investigation (lead); Project administration (equal); Visualization (lead); Writing—original draft (lead); VS: Investigation (supporting); Visualization (supporting); ME: Conceptualization (equal); Funding acquisition (lead); Project administration (equal); Writing—original draft (supporting); Writing—review & editing (lead).
